# You are my death: the shattered temporalities of zombie time

**DOI:** 10.12688/wellcomeopenres.15966.1

**Published:** 2020-06-10

**Authors:** Martin O'Brien

**Affiliations:** 1 Department of English and Drama, Queen Mary, University of London, London, E9 6DW, UK

**Keywords:** Death, Zombie, Cystic Fibrosis, Performance, Art, Time, Cough

## Abstract

This essay considers the relationship between the experience of life shortening chronic illness and the current COVID-19 crisis. Martin O’Brien uses his experience of living with cystic fibrosis to interrogate the temporal experience of living within a global pandemic. He returns to his concept of zombie time, the temporal experience of living longer than expected, in order to understand the presence of death as a way of life. The essay uses some of O’Brien’s own art practices, and an analysis of his own sick, coughing body in order to think through what it means to live with cystic fibrosis during a pandemic, which mimics much of its features. O’Brien argues that we are currently occupying a widespread zombie time, which frames other people as carriers of death, and that we must find ways of being together in order to survive.

*This used to be the most optimistic city in the world. Now the sun never rises. The shops are never open. The birds never sing. The streets are always empty. Cars lie abandoned, Buildings crumble or stand as empty shells. This city is full of places where flowers don't grow, gravel and dirt places, underpasses, places that look as though they’ve come straight from a horror film, places that are always empty, places that seemed to be made to walk through at night, places you would never take a first date, long passages, tunnels, non-places, dilapidated places, places where you would expect to be taken by a murderer. Perhaps this place used to be beautiful but this place seems as though it has experienced the apocalypse*.
                                                                                                                                           Text from *The Unwell*, 2016

My artist studio is full of coffins at the moment. They are stacked up on top of each other, still covered in bubble wrap. There are nine of them in total. These were to be the materials for my new performance *The Last Breath Society (Coughing Coffin)* at the Institute of Contemporary Arts (ICA), London. The work was due to be performed at the end of March. It was exploring ideas of community, closeness and loneliness in sickness and dying. This work couldn’t happen but seems more relevant now than it has ever been. A few weeks ago, as the new pandemic ravaged throughout the world, I called a friend who was involved in the performance to let her know that it was cancelled. The first thing she said was ‘Martin, it’s like the whole world has caught CF’. I was diagnosed with cystic fibrosis (CF) when I was six weeks old, and my friend is the artist Sheree Rose, whose partner, Bob Flanagan, died from CF in 1996. Her assertion was startling for me, and I started to think about the experience and aesthetics of the pandemic, and the ways in which they mimic a life lived with CF. I began to think about the ways in which the experience of CF management, and our peculiar survival strategies were being taken up by a wider population. Gloves and masks are a regular part of CF experience, and social distancing is a very familiar term for anyone with the disease, as I will discuss in this writing. The symptoms of COVID-19 are also part of CF experience: coughing, shortness of breath, and exhaustion.

This piece of writing is an attempt to do two things. Firstly, to use CF experience to think through the current pandemic and tease out what a wider population might understand about illness, mortality, and isolation by looking at life lived with CF; secondly, to consider what it means to be sick in a time when everyone is suddenly becoming aware of their own mortality.

Growing up with CF means a constant facing of your own mortality. My older cousin died from the disease aged 12, when I was eight years old. This was the first time I understood death as part of life. This was the first time I knew that I would not live to see my hair turn grey. This was the first time I began to understand existence and temporality under the conditions of disease. The life expectancy for someone born in 1987 with CF is 30 years old; this information was plastered all over charity appeals for the CF Trust. I was sure I would die at 30. The temporal movement towards this age was the defining condition of growing up for me. Death was an obsession. I reached and surpassed 30. Death did not come for me. In attempting to understand what it means to live longer than expected, I formulated the notion of zombie time. This is the temporal experience of living on when death was supposed to happen. I have previously written about zombie time as being:

[…] a different relationship to death and life […] It’s a form of enduring life when death is no longer the certainty it once was. It is no longer linear, it’s full of breaks and ambushes. In zombie time, you keep moving but not towards anything, just for the sake of moving. No goals, only desires. No plans, only reactions. The only constant is the presence of death but not in the way it once was, for the zombie knows death and breathes in death. Death is in me, instead of somewhere else.
                                                                                                                                           ( O’Brien & Bouchard, 2019: 263)

Zombie time offers a way of conceptualizing a changing relationship to mortality. The temporal experience in my childhood and early adulthood was one of moving towards a death date. As Lauren Berlant would have it, it is ‘the embodiment toward death as a way of life’ ( Berlant, 2011: 114). Zombie time insists on a different temporal proximity to death. Like the Hollywood zombie which holds within it a paradox, in that it is both dead and alive, those of us living in zombie time experience death as embodied in life, rather than Berlant’s movement towards death. We had come to terms with the fact that we are about to die, and then we didn’t. This necessitates a fundamental change in how we imagine death and our position in relation to it. In *The Birth of the Clinic*, Foucault proposes that the sick living body is the anticipation of the corpse it will become ( Foucault, 1973: 162). Perhaps, though, zombie time offers us a way of conceptulising sick life, not as an anticipation, but as already a corpse, one with new life breathed into it. The end of life is now ambiguous. Death is keenly felt as lived experience but not as something in an imagined future, rather something we are constantly living through. There is no grim reaper anymore; death is not external but exists within a person, as the experience of living. I want to suggest that the temporal experience of living in this pandemic might be something akin to zombie time in that it is necessitating a changing relationship to sickness and mortality for large portions of the population. As I will argue, the virus means that the previously healthy are thrust into a contiguity with sickness and death, which means they are forced to face their own mortality. Zombie time helped me understand my own experience of temporality and the way in which death functioned as part of life. It offered an articulation of something which I was unable to find elsewhere. Perhaps it might be useful for trying to describe the temporality of the pandemic and the ways in which people are forced to become acutely aware of death as part of their own life. There is a wide spread imagination of what death might look like for each individual and our families. It seems as though we are living in a temporary global zombie time, one which shatters the temporalities of healthy living. These shards of temporal experience may well be put together again, but for now we exist in a liminal temporal space. The imagined futures are being changed fundamentally and much rhetoric is around surviving this crisis, ‘no goals, only desires, no plans, only reactions’ ( O’Brien & Bouchard, 2019: 263).

*The Last Breath Society (Coughing Coffin)* is indicative of much of my practice. My work has consistently used the materiality of my disease (breath, mucus, coughing) to explore what it means to be born with a life shortening illness. I had often insisted on the isolation of dying young, as a lonely process, as one which renders a person outside of the dominant experiences of life. Indeed, the experience of living with CF can be solitary. Those of us with the disease are unable to be in a room with any others who share the condition. We must remain six feet apart from one another when outdoors. Those who best understand the *feelings* of CF are unable to be together, unable to talk about survival strategies, unable to hug, kiss or have sex. But my feelings about the loneliness of sickness and dying are changing. The Last Breath Society is, in some ways, a gathering of my horde.

The Last Breath Society is a semi-fictional group. It is a gathering of those living in zombie time and others who are forced to consider what death is because of their proximity to it. Perhaps now, my neighbors and co-workers should all be part of the society. Perhaps now, the politicians, who underfunded our health service, and the footballers should be part of the society. Perhaps now the doctors and post people, the nurses and the drag queens, the police and the deep-sea divers, should all be part of the society. The explorers and the singers, the cleaners and the stadium announcers should join the society. The young should now join the old in the Last Breath Society. Perhaps you should be part of the society. The grim reaper stands outside all of your doors now, and there is no way to ignore the knocking. His skeletal face peers through your window and watches you sleep, and it’s terrifying. He has become a friend to me, but a friend that will one day betray me. I know not to trust him.

*Time has ended here. The sun has vanished. Everything remains as it was but now the pavements and subways lie silent. The shops are full of dog food but there are no dogs to eat it. The machines still run but they no longer serve a purpose. The lights remain on, burning bright but they will eventually fade to darkness*.
*But something still remains here. There is movement in this city. Something moves slowly through the darkness. They have replaced human life. They resemble us but they are not us. They fill the city with an unwell sound.*
                                                                                                                                           Text from *The Unwell*, 2016

As an artist, my work has often imagined worlds in which only the sick can survive. My film, made in collaboration with Suhail Merchant, *The Unwell* was shot in 2015 and first shown in 2016. It is set in a city, in which it is always night, human life has been replaced by the unwell. These are B-movie style zombies are always seen alone (I play all of them) staggering through an empty city, coughing. The opening of *The Unwell* is a series of empty streets, roads and buildings at night. Watching it back now, seems eerie, as I look out of my window at the empty London streets. The film imagines a new form of life, we never see how the city became over run by zombies, just the aftermath. As such, very little happens. The film uses the aesthetics of a dystopian apocalypse. Now though, as we enter into a strange shattering and reforming of our everyday, the figures in the film might be read as standing in for all of us who exist in zombie time. Unlike *The Last Breath Society (Coughing Coffin)*, which is attempting to bring people together, to combat the loneliness of temporalities that put barriers in the way of closeness, *The Unwell* demonstrates the distance between bodies and experiences.

There is never more than one unwell figure in any shot. They stand or walk alone, solitary and ambling, coughing and bleeding. In my work, the cough is a symbol of hope, a symbol of the future and a symbol of change. The cough functions as a sick language of sorts, as these unwell beings speak out to no one but themselves. The coughs become a soundscape of the city, ringing out through the night. I want to think about the figure of the solitary cougher, and its importance in understanding zombie time. In essence, this is the enduring image of zombie time. The lonely figure, coughing, afraid of infecting others or making life worse for themselves. But how might we become The Last Breath Society? How can we stand together in zombie time?

As someone with CF, I have coughed all my life. Although my regular coughing fits have often caused worry in public places, never have I so keenly felt the disgust and fear towards me as during the beginnings of the spread of COVID-19 in London. Simon Bayly suggests that the ‘cough is the “creature voiced”, but also what molests the vocal organs, barely fit for thought, let alone philosophy. Philosophy has sought to erase the cough, to eradicate its interruptive force’ ( Bayly, 2011: 166-167). He highlights ‘Aristotle’s rejection of the cough as merely the impact of the breath’ (ibid: 167) and Husserl’s ‘consignment of all paralinguistics or kinesis expression to the category of the meaningless’ (ibid: 167) as examples of ‘philosophy’s repulsion for the organic process of vocalization’ (ibid: 167). It is difficult to ignore the philosophical importance of the cough now, though. As one of the most common symptoms of COVID-19, a focus on the cough has become one of the enduring legacies of the pandemic. For Steven Connor ‘the cough is voice coerced by breath, not breath tuned and tutored into voice.’ ( Connor, 2007). The cough functions as a form of non-propositional language, this is a reflexive, interruptive language, in which ‘the air is not expressed, pressed out into audibility, impressed into audible shapes and postures, but seems rather to be escaping, as though through a rent or gash’ ( Connor, 2007). David Appelbaum suggests that a ‘cough is the detonation of voice’ ( Appelbaum, 1990: 2). I have explored the philosophy of the cough elsewhere, in which I develop Appelbaum’s work by thinking through the ways in which the cough functions as a vocalisation of illness:

If the cough is the detonation of voice, though, it is equally the forceful establishment of a different voice, one which does not adhere to language- the voice of illness. The cough interrupts, it is something that cannot be contained and demands its right to be heard. It functions as a disordering of the voice and of the breath.
                                                                                                                                           ( O’Brien, 2016: 132)

Zombie time is constantly ruptured by the excessiveness of coughing fits. They function as a marker of sickness. Appelbaum continues thinking about the nature of the cough:

It is duller than the pierce of a cry which goes to the heart. On the terminal ward, one hears the cries first. But the coughs penetrate more deeply, into the compact soma of the body. There they contact an organic memory which reminds us of death and life as facts unembellished by feelings. If the world were cured of the common cough, we would be less prepared for our earthly passage.
                                                                                                                                           ( Appelbaum,1990: 2)

If the cough ‘reminds us of death and life’ (ibid: 2) then it is something to be avoided as it reminds us of the potential of death within life. The sound of the cough ‘seems to initiate our deepest bodily identification. It is as if the cough speaks directly to the flesh of others, like a warning siren, triggering bodily memories of illness’ ( O’Brien, 2016: 132). Just as Foucault discussed the living sick body as anticipation of corpse, Appelbaum thinks of the cough as preparation for death. Somehow, in the sound of the cough lies the memory of death. The cough is an opening into zombie time. It ruptures our stable temporal experiences, and thrusts us into the peculiar shadow temporality of zombie time.

For someone with CF, the cough has always been a marker of identity. I can recognise a CF cough anywhere, the raspy, moist, phlegm filled sound which vibrates through the floor. The cough of another CF sufferer has a strange impact, a sense of shared experience with another, but also fear. This cough has the potential to make me very ill. But now, in the zombie time of COVID-19, the cough represents the virus and acts as a reminder of mortality, not just for the cougher but for all who hear it too. The cough is now synonymous with the virus. It is a reminder of the dangers of the outdoors, of surfaces, and more significantly, the dangers of other humans. Your own death is potentially in the lungs of another. The cougher holds your mortality in their chest, and you hold theirs in yours.

In *The Last Breath Society (Coughing Coffin)*, which we are still waiting to perform, people will enter into a dark space. The opening image will be a series of coffins, closed, laying on the ground. From within them, the sound of coughing emerges. The coffins are sealed shut, and the bodies are inside. In this instance though, the cough serves as a confirmation of life. The corpse does not cough. If the coughing body acts as a *momento mori*, it is also a reminder of breath, and of life. The last Breath Society is about coming together to remember we will die, but it is also a celebration of life, a defiant gathering for the sake of survival.

*They stand on two feet and wear our clothes but they are not us. Do they eat and sleep? Do they dream or even recognise their own reflections? Their actions seem to serve no purpose, they amble through this urban wasteland coughing and spluttering. Their steps are laboured and clumsy. Their garments are covered in blood, the faces with great wounds and then the eyes. They witness but they do not comprehend, they are blank and without personality. The empty eyes gaze straight ahead but towards what future? What do they remember? Their coughs ring out all over the city. These bodies are like factories, mass producing mucus. They seem to breathe but we don’t know if their hearts beat. Do they have the capacity to learn? To feel emotions? The only thing we can be sure of is that they are profoundly unwell*.
*They wear clothes that could define them but whoever they used to be no longer matters. They are simply unwell. They move alone but together they inhabit the entire city. Do they interact with one another? Are they ever lonely? Do they understand the nature of their existence? The meaning of all of this? Do they have the capacity to love? To hate? They smell like death. They thrive in the dark, they thrive in the cold. They know no masters. They own this city which was once ours*.
                                                                                                                                           Text from *The Unwell*, 2016

In both CF and in the time of COVID-19, closeness is prohibited. Over the last 20 years or so, research into cross infection in CF has meant that I should avoid anyone else with the disease. All cultural representations of CF have focused on this aspect of the illness in recent years. There is an episode of the popular American hospital-based television series *Grey’s Anatomy* ( American Broadcasting Company, 2011) based on a patient with CF. He is a young man who comes into hospital for a lung transplant. The doctors soon discover that his girlfriend also has CF. The doctors say he is ‘committing suicide’ and they will not perform the lung transplant unless he and his girlfriend break up as he would be ‘wasting the lungs’. In 2019 Hollywood did CF with the film *Five Feet Apart*: ( Baldoni, 2019). It told the story of two CF sufferers who fall in love, they break the six feet apart at all times rule by ‘stealing a foot’. These two examples are soppy, romanticised versions of sickness, and the tragedy of separation plays so well into the Hollywood trope of forbidden love. However, they do highlight something significant. The characters in these two fictions are looking for connection with someone *like them*. The disease prevents them from having a physical relationship. Remembering my own childhood, before cross infection was discovered, playing with the other children with CF in the hospital reminds me of the comfort of being around others that understand. It is startling to watch footage in the documentary about Bob Flanagan and Sheree Rose, *Sick: The Life and Death of Bob Flanagan, Supermasochist* ( Dick, 1998), in which Flanagan would be a leader for an annual camping trip for children with CF. The footage shows them sat together around the camp fire singing, creating community through physical closeness.

The implication of our inability to be together is that in our violent CF coughs there exists the potential for harm towards another. On our fingers, and even in our breath, there exists dangerous bacteria that might make someone else very ill or even shorten their life. In staying away, we are helping someone else but also helping ourselves. Physical closeness is craved but dangerous. In her book *Cruel Optimism*, critical theorist Lauren Berlant suggests that ‘[a] relation of cruel optimism exists when something you desire is actually an obstacle to your flourishing.’ ( Berlant, 2011: 1). Closeness in CF is a form of cruel optimism. This can be extended to understand the current situation. We long to be together with friends and loved ones, but that closeness that we desire is not simply an obstacle to an individual flourishing but to a population battling to survive a pandemic.

Now the position of The Last Breath Society seems significant. How can we be together when we clearly cannot be together? The multitude of online options cannot replace touch, or closeness. Zombie time holds within its nature a cruel optimism. We are united through a temporal experience but cannot form important and necessary friendships, or communities. Our CF bodies, which are failing, are left to do so alone. The people we worry about as the virus spreads, are disembodied voices on the end of a phone. What we would give to hold the people we love now. What we would give to love the people that share our experience.

The zombie time of COVID-19, as well as CF, frames the other as danger, as carrier of your death. It also imposes upon you the responsibility of other people’s lives. That is the impossibility of closeness in times of infection. The shattering of temporal experience means that zombie time is defined in relation to our own mortalities and the place of others in this. Inherent within the make-up of zombie time is the need for survival, to continue. The zombie is driven only by the desire to survive, both as an individual and a species. The zombie knows nothing but desire for human flesh; it bites in order to feed and this produces more zombies. Zombie time is living with death inside you, and that’s what we are all doing now. So, welcome to The Last Breath Society, a place where we can decay together.

*This used to be the most optimistic city in the world. Now it’s full of darkness illuminated by the fading street lamps. Out of this darkness stumbles life quite different from us. The unwell negotiate this landscape in a way we could not. There is no war in this city, no poverty, no crime, nothing to fear. There is only sickness and this sickness is itself a form of existence, a way of seeing and being, a way of breathing and moving. This is life. They do not fear death because death is already behind them. They are not motivated by material things. To witness the unwell is to understand all of our fears but our fears mean nothing to them. This city used to be our future but now the future belongs to the unwell.*
                                                                                                                                           Text from *The Unwell*, 2016

## Data availability

All data underlying the results are available as part of the article and no additional source data are required.

## Author information

Martin O’Brien is an artist, thinker, and zombie. He works across performance, writing and video art in order to examine what it means to be born with a life shortening disease. His writing also reflects on the experience of illness and the ways in which other artists have addressed it. A book of writings about Martin, *Survival of the Sickest: The Art of Martin O'Brien* was published in 2018 by the Live Art Development Agency. His performance work has been shown throughout the UK, Europe, US, and Canada. His writing has been published in books and journals on performance, art, and the medical humanities. Martin is currently lecturer in Performance at Queen Mary University of London.
