# Supplementary material for: You are my death: the shattered temporalities of zombie time
Source: Wellcome Open Res. 2020 Jun 10;5:135. [Version 1] doi: 10.12688/wellcomeopenres.15966.1 (PMC7324942; doi:10.12688/wellcomeopenres.15966.1)
Supplement: Peer review history

# Peer review: Reviewer response for version 1

**DOI:** 10.21956/wellcomeopenres.17510.r39021

**Published:** 2020-06-29

**Authors:** Alan Bleakley

**Affiliations:** 1 Faculty of Health, Peninsula School of Medicine, Plymouth University, Plymouth, UK

The criteria for scientific papers do not apply – this is a discursive arts piece (and welcome).

 I have followed Martin O’Brien’s work for some time and he is amongst the foremost performance artists and commentators globally who have turned their symptoms into their life’s work as a commentary on the pathologizing of natural phenomena. Martin literally embodies a ‘third state’ of being between life and death that challenges traditional conceptions of temporality, as a suspended ‘zombie’ time. This article puts his work into the context of the fears of pathologizing brought by the current coronavirus pandemic. He argues, originally, that this crisis forces us to re-think what it is to live together contra optimistic models (I am thinking of Roland Barthes’ How to Live Together, and Ivan Illich’s Tools for Conviviality).

 Martin points to the uncanny resemblance between Covd-19 behaviour and CF precautions, that can be read as a performance piece in itself, partly scripted by ‘the science’, but improvised and moulded by circumstance: wearing gloves and mask, distancing for fear of catching an infection. This is living in “zombie time” – but this is neither suspension of time, ‘flatlining’, nor emptiness; it is, rather, full of “breaks and ambushes” – sudden, startling surprises. My thought when I first read this was: ‘this is not living in a bubble, but in a hedge, a thicket, a tangle of thorns’. This is thanks to the literary element in Martin’s writing – an idiosyncratic mix of tragic and lyrical genres.

 More, besides offering an insight into a different way of living in time, Martin also describes an identity construction that offers resistance to ‘medicalizing’ - that fetishizes life at all costs. In contrast is the “zombie time” that Martin describes as ‘dystopian’, but I think he means ‘atopian’ – a-topos, a ‘nowhere’ space. Yet, as noted above, this is a space full of “breaks and ambushes”. But I must clarify this observation, where Martin makes the distinction between, say, a performance space with a public, and the contradictory ‘meeting space’ of CF sufferers only, where ‘meeting’ is impossible in terms of intimacy, tenderness, skin-to-skin, breath-to-breath contact. Here, in the CF community, people are literally ‘passing through’. But through the idea of a common “The Last Breath Society”, we are, in Covid-time, all embraced by “zombie time”. This is an important and exceptional model of empathy or what Hardt and Negri call “the commons” (a common wealth as a properly democratic world).

 In Martin’s a-topian vision, “the cough” becomes chief signifier, and here Martin has contributed greatly to semiotics in the tradition of Roland Barthes, where universal embodied signs (coughs, phlegm, bodily fluids) become both symbols and concrete language. (p.4 on has a brilliant phenomenology of the cough, developing a previous theme in Martin’s work). In zombie-time, the greeting is the cough and splutter. Paradoxically, what frightens people and keeps them apart in Covid-time – and why we are masked against it – serves precisely the opposite function in zombie-time: of greeting, bonding and signification of a common love (“the cough becomes the soundscape of the city”). Perhaps, as in China Miéville’s The City and the City, two cities – ‘the well’ and ‘the sick’ – are superimposed on each other, so that citizens live side-by-side but are forbidden by law and strict censure, to not acknowledge or even glance at each other. To do so can mean being infected and dying at the hands of the Police who control the ‘borders’. More, each city has an idiosyncratic aesthetic. Each city then retains its identity through the coded ways in which it rejects the existence of the other city. I see this same idea running through Martin’s work.

 The piece is absorbing and focused, with original contributions to the literature. My only suggestions are that I would like to see some comment on politics. Just as Covid-19 ‘science’ has become politicized, so “zombie time” surely asks questions about the relationships between illness and power. Those with CF are experts in their own conditions and thus form active resistance against ‘medicalizing’. Just look at Martin’s own power to diagnose his own state of being through the body, just as medical students are taught to palpate, percuss and auscultate. Martin’s descriptions show Foucault’s ‘biopower-as-resistance’ in full flow.            

 Further, it's not just somatic suffering that creates “zombie time” or suspended states, but many mental illnesses do this too – what are the corresponding mental states to bodily suspension as zombie?

 Finally, Giorgio Agamben’s idea of zoe, or bare life (rather than bios, or a ‘qualified’ or full life affording an identity), as an excommunication, a prisoner of war, an outcast (leprosy), and so forth, is worth exploring in relation to “zombie time” as an identity.

 Timely and extraordinary piece of writing.       

Is the work clearly and accurately presented and does it cite the current literature?

Yes

If applicable, is the statistical analysis and its interpretation appropriate?

Not applicable

Are all the source data underlying the results available to ensure full reproducibility?

No source data required

Is the study design appropriate and is the work technically sound?

Yes

Are the conclusions drawn adequately supported by the results?

Yes

Are sufficient details of methods and analysis provided to allow replication by others?

Yes

Reviewer Expertise:

Psychological medicine, medical education, medical humanities

I confirm that I have read this submission and believe that I have an appropriate level of expertise to confirm that it is of an acceptable scientific standard.

# Peer review: Reviewer response for version 1

**DOI:** 10.21956/wellcomeopenres.17510.r39020

**Published:** 2020-06-15

**Authors:** Amelia Jones

**Affiliations:** 1 Roski School of Art and Design, University of Southern California, Los Angeles, CA, USA

Let me say that all of the questions on the Wellcome form about “data” are not relevant. This is a humanities/arts essay beginning with the personal experience of suffering from Cystic Fibrosis and extending this experience to the current COVID-19 situation. There is no call for outlining of “methods” (other than O’Brien’s very clear statement of the premise and goals of the article on page 2 of the PDF), nor, in this kind of research/writing, are “conclusions drawn” to be considered “adequately supported by… results.” This kind of work is fluid, poetic, but deceptively simple in that there is a strong underlying argument about our relationship to death, which is compellingly played out throughout with the author’s personal examples as well as some citations of (highly appropriate) theorists who have written about illness, death, or related topics.

 This is a wonderful, deeply moving essay. Nothing major needs fixing or changing, with possible exception of the larger suggestion below. The essay is a textured exploration of our current global situation that toggles successfully between stories of O’Brien’s experience as a CF sufferer and COVID-19’s viral reminder of potential imminent mortality.

 My one larger suggestion: Given the massive protests for Black Lives Matter globally but especially in the US in the past weeks (certainly after O’Brien would have finished this essay), it feels odd in making any revisions not to deal with the specific issue of what kinds of people are more vulnerable to COVID-19. And is this paralleled by CF? Are less advantaged people, or certain ethnic groups, more likely to inherit the disease? I’d guess economic class is not relevant for CF, but it would be interesting to raise these points. People of color in the US, due to well document “weathering” for African Americans in particular, the lower quality of health care for POC in general (in the US especially), and the likelihood of POC being forced to continue working in proximity with others in blue-collar jobs. POC in the US and UK (and probably beyond) are dying in vastly disproportionate numbers to white middle-class folks, from what I have read. I mention this because the initial terror we all felt has dropped off for many white middle-class people with the increasingly apparent advantage we have in avoiding dying of COVID-19. So the parts of the essay addressing our fear in fact apply more to O’Brien and to those of lesser economic status in places like the UK and US than they do to white people such as myself with fewer or no pre-existing conditions. This shifts the overall feeling—palpably in the last two weeks in the US, we have (absolutely rightly) all come to be more aware of these disparities.

 Vis à vis the phrase “memory of death”: this is extremely provocative as an idea and could be elaborated. That is, most of us think about the “anticipation” of death, not a “memory” of it (which would imply it occurred already in the past). O’Brien could connect this memory of death idea to zombie time—doesn’t it activate a kind of scrambling or reversal of temporality, which he suggests is the experience of CF sufferers?

Is the work clearly and accurately presented and does it cite the current literature?

Yes

If applicable, is the statistical analysis and its interpretation appropriate?

Not applicable

Are all the source data underlying the results available to ensure full reproducibility?

No source data required

Is the study design appropriate and is the work technically sound?

Yes

Are the conclusions drawn adequately supported by the results?

Yes

Are sufficient details of methods and analysis provided to allow replication by others?

Yes

Reviewer Expertise:

Art history, cultural studies, performance studies, gender/sexuality studies

I confirm that I have read this submission and believe that I have an appropriate level of expertise to confirm that it is of an acceptable scientific standard.
